# Natural Dietary Compounds in the Treatment of Arsenic Toxicity

**DOI:** 10.3390/molecules27154871

**Published:** 2022-07-29

**Authors:** Geir Bjørklund, Md. Shiblur Rahaman, Mariia Shanaida, Roman Lysiuk, Petro Oliynyk, Larysa Lenchyk, Salvatore Chirumbolo, Christos T. Chasapis, Massimiliano Peana

**Affiliations:** 1 Council for Nutritional and Environmental Medicine, Toften 24, 8610 Mo i Rana, Norway; 2 Department of Environmental and Preventive Medicine, Jichi Medical University School of Medicine, Shimotsuke 329-0498, Japan; shiblu@jichi.ac.jp or; 3 Graduate School of Environmental Science, Hokkaido University, Sapporo 060-0810, Japan; 4 Department of Pharmacognosy and Medical Botany, I. Horbachevsky Ternopil National Medical University, 46001 Ternopil, Ukraine; shanayda@tdmu.edu.ua; 5 Department of Pharmacognosy and Botany, Danylo Halytsky Lviv National Medical University, 79010 Lviv, Ukraine; pharmacognosy.org.ua@ukr.net; 6 CONEM Ukraine Life Science Research Group, Danylo Halytsky Lviv National Medical University, 79010 Lviv, Ukraine; 7 Department of Disaster Medicine and Military Medicine, Danylo Halytsky Lviv National Medical University, 79010 Lviv, Ukraine; petrolinik1@gmail.com; 8 Department of Chemistry of Natural Compounds, National University of Pharmacy, 61002 Kharkiv, Ukraine; larysa.lenchyk@nuph.edu.ua; 9 CONEM Ukraine Pharmacognosy and Natural Product Chemistry Research Group, National University of Pharmacy, 61002 Kharkiv, Ukraine; 10 Department of Neurosciences, Biomedicine and Movement Sciences, University of Verona, 37134 Verona, Italy; salvatore.chirumbolo@univr.it; 11 CONEM Scientific Secretary, strada Le Grazie 9, 37134 Verona, Italy; 12 NMR Facility, Instrumental Analysis Laboratory, School of Natural Sciences, University of Patras, 265 04 Patras, Greece; cchasapis@upatras.gr; 13 Department of Chemical, Physics, Mathematics and Natural Sciences, University of Sassari, Via Vienna 2, 07100 Sassari, Italy

**Keywords:** arsenic, toxicity, natural compounds, vitamins, trace elements, medicinal plants

## Abstract

Chronic exposure to arsenic (As) compounds leads to its accumulation in the body, with skin lesions and cancer being the most typical outcomes. Treating As-induced diseases continues to be challenging as there is no specific, safe, and efficacious therapeutic management. Therapeutic and preventive measures available to combat As toxicity refer to chelation therapy, antioxidant therapy, and the intake of natural dietary compounds. Although chelation therapy is the most commonly used method for detoxifying As, it has several side effects resulting in various toxicities such as hepatotoxicity, neurotoxicity, and other adverse consequences. Drugs of plant origin and natural dietary compounds show efficient and progressive relief from As-mediated toxicity without any particular side effects. These natural compounds have also been found to aid the elimination of As from the body and, therefore, can be more effective than conventional therapeutic agents in ameliorating As toxicity. This review provides an overview of the recently updated knowledge on treating As poisoning through natural dietary compounds. This updated information may serve as a basis for defining novel prophylactic and therapeutic formulations.

## 1. Introduction

Arsenic (As) is a metalloid, which is the chief among the dangerous toxins of the environment, currently present in the groundwater of some territories (Bangladesh, India, Taiwan, Mexico, etc.) [1]. It is also widespread as an industrial pollutant [2,3,4,5]. Arsenic is naturally found in groundwater, and due to the dissolution of As-containing minerals, many people can be exposed to its toxic effect after continuously using contaminated water [1,6]. Arsenic and fluorine (F^−^) are considered the two most widespread contaminants in drinking water, causing detrimental effects on public health in many countries worldwide [7]. The use of As in the industry has led to its extensive environmental spread, further increasing its negative effects on human health. A significant source of human exposure to As comes from its natural levels in vegetables, fruits, grains, cereals, dairy, and meat [8]. 

Furthermore, if these foods are produced in naturally rich or As-polluted areas, the accumulation of As in food increases considerably [9]. Rice and grain plants can absorb arsenic more easily than other vegetables. Some varieties of fish, shellfish, and even seaweed can contain high levels of organic arsenic, which is less toxic. Long-term exposure to As through ingestion, dermal contact, or inhalation can provoke multisystem health abnormalities such as cardiovascular and blood diseases, neuro- and nephrotoxicity, dermatitis, and several types of cancer [2,10,11,12,13,14]. However, chronic As toxicity produces various health problems, and the diagnostic criteria considered for chronic arsenicosis are dermal manifestations such as hyperpigmentation and hyperkeratosis [15]. More than 200 million people worldwide are currently exposed to its chronic effects [2,16].

Due to the extensive damage of various body organs caused by As, investigations of therapeutic methods for its treatment are current and important issues. Measures have urgently required that focus on reductions in As toxicity, early diagnosis, and therapy of As-induced diseases.

Avoiding the consumption of water contaminated by As is the first recommended step to counteract the phenomena of arsenicosis [7,17,18]. Chelation therapy is the most widely used method for arsenicosis treatment, but it is associated with hepatotoxicity, neurotoxicity, blood abnormalities, and other adverse effects [19,20,21,22,23]. Phytopreparations and other natural products can effectively relieve As-mediated toxicity without particular side effects [24].

Since As affects the intracellular antioxidant machinery, exogenous supplementation of antioxidants can counteract the pro-oxidant stress induced by As [25,26]. The treatment options advocated are vitamins and mineral supplements and antioxidant therapy [26].

Antioxidants are also recommended as a symptomatic treatment since the metabolism of As in the body can increase the generation of free radicals and provoke oxidative stress [27,28]. Nutrition is crucial in preventing and developing As-related disorders [27,29]. Low dietary intake of protein and micronutrients increases susceptibility to As-related diseases [29,30]. This could be because nutrition deficiency results in the slow removal of As from the body. A properly selected diet and nutrition can positively affect the body’s metabolism and reduce the toxic effects of As [29].

Many natural dietary compounds have been found to exhibit antioxidant properties that are beneficial in treating the toxic effects of As. The positive influence of vitamins (A, C, and E), polyphenols, and curcumin, which regulate the activity of glutathione and antioxidant enzymes (catalase, superoxide dismutase, and glutathione peroxidase) in their protective roles against oxidative stress caused by As, has been established [31,32]. The high content of hydrophilic phenolic compounds in herbal extracts and aqueous infusions can provide notable antioxidant effects [33,34]. For instance, black and especially green tea polyphenols significantly reduce As-induced toxicity in experimental animals [35]. Exogenous antioxidants such as the microelements zinc and selenium are also very useful for As detoxification [36,37]. This review aims to describe the main cellular targets vulnerable to As compounds and provide an updated overview of the knowledge about the treatment/intervention for As poisoning through natural dietary compounds. This updated information may serve as a basis for defining novel prophylactic and therapeutic formulations.

## 2. Main Cellular Targets Vulnerable to Arsenic Inhibition

Underlying the mechanism of As toxicity is its binding with important redox regulators and signaling and DNA repair proteins. In particular, vulnerable targets for the toxic action of inorganic iAs^III^ and organic As (i.e., monomethylarsonous acid MeAs(OH)_2_, MMA^III^, and dimethylarsinous acid Me_2_AsOH, DMA^III^) are the thiol and selenol groups of crucial antioxidant, defense, and ROS scavenging enzymes, whose inhibition explains the oxidative stress, cell damage, genome instability, and carcinogenesis associated with chronic arsenic exposure [38]. Arsenic can directly replace essential zinc ions in the important metal binding sites of proteins and/or induce reversible and irreversible oxidative and nitrosative modifications of crucial cysteine residues due to ROS/RNS generation, leading to zinc release and protein conformation modification, with subsequent function inhibition [39,40]. Indirect oxidative damage due to the overproduction of ROS also occurs in lipids(peroxidation), proteins (carbonylation, misfolding, and epigenetic dysregulation), and DNA (strand breakage) [41,42]. In the following sections, an overview of the main target proteins of As is described.

### 2.1. Pyruvate Dehydrogenase

In the mitochondria, pyruvate dehydrogenase (PDH) catalyzes the oxidative decarboxylation of pyruvate to CO_2_ and acetyl-CoA, which is then oxidized in the TCA cycle to produce energy.

During World War II, Rudolph Peters and his group identified PDH (in those days termed the target for ‘the biochemical lesion’) as a target for As (from the arsenical poison war gas, Lewisite) [43]. In this enzyme, the vulnerable target is the cofactor lipoic acid, or its dithiol form, dihydrolipoic acid (DHLA).

**Figure 1:**
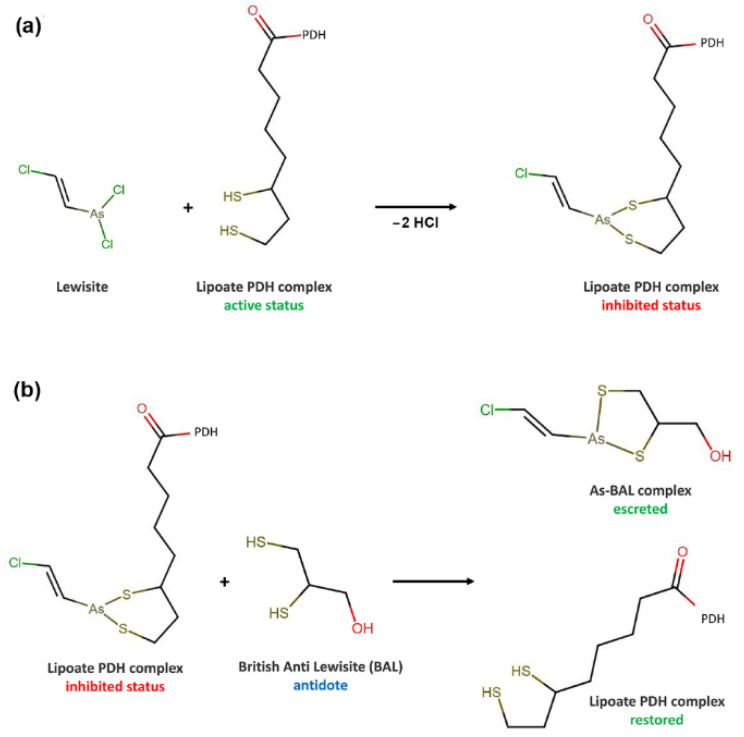
(**a**) Mechanism of Lewisite interaction (and similarly, monomethyl As(III) acid) with lipoic acid and the subsequent inhibition of PDH activity; (**b**) the antidote action of BAL and the restoring of PHD function.

During the intoxication, two Cl atoms of Lewisite can be replaced by the two sulfur groups of the dithiol DHLA since As has a higher affinity to sulfur than to chlorine, the PDH enzyme activity is blocked, and the production of acetyl-CoA for the citric acid cycle will be completely inhibited (Figure 1). The specific synthesized antagonist As-chelating agent can restore the PHD activity; British Anti-Lewisite (BAL) competes with protein thiol groups for As binding. Once complexed, complexed is then excreted in the urine [44].

The most toxic metabolite of inorganic As(III) is MMA^III^, which has one methyl group and two -OH groups attached to the As core [45]. Since two sulfur groups of DHLA have a higher affinity than oxygen to As, it is reasonable to assume that the same enzyme is attacked through the same cofactor.

### 2.2. Glutathione and Glutathione-Related Enzymes

In vitro studies on the effect of arsenic compounds on glutathione-related enzymes have shown that arsenic compounds destroy cellular antioxidant defense mechanisms by consuming glutathione (GSH) and inhibiting the enzyme responsible for its recycling [46,47,48]. Redox imbalance due to the As exposure occurs, with the direct alterations of antioxidant enzyme function such as glutathione reductase (GR), superoxide dismutase (SOD), glutathione peroxidase (GPX), catalase (CAT), and glutathione S-transferase (GST) [46]. GR is a flavoprotein vulnerable to low dietary intake of riboflavin [49], which catalyzes the reduction of glutathione disulfide (GSSG), restoring intracellular GSH with the involvement of nicotinamide adenine dinucleotide phosphate (NADPH) as an electron donor [50]. GR is of fundamental importance in the antioxidant defense and detoxification process. In fact, during the oxidative stress condition, excessive ROS production, and SOD and CATs are activated to produce lipids and hydroperoxides. Se-dependent GPX detoxifies hydroperoxides to their corresponding alcohols and free hydrogen peroxide to water, with GSH acting as an electron donor, producing GSSG as a final product. Then, GR catalyzes the reduction of GSSG to restore GSH. Arsenic exposure enhances oxidative stress by the depletion of GSH and the impairment of ROS-scavenging enzymes [46,51]. In the endoplasmatic reticulum (ER), where the GSH system is the primary redox buffer, the depletion of GSH disrupts protein quality control leading to protein misfolding [42,51].

### 2.3. Thioredoxin and Thioredoxin Reductase

The thioredoxin system, comprised of NADPH, thioredoxin reductase (TrxR), and thioredoxin (Trx), functions as an important part of the cellular antioxidant defense [52]. It exerts its activities via a disulfide-dithiol exchange reaction. TrxR, together with Trx, plays a crucial role in restoring oxidatively damaged proteins by reducing abnormal protein disulfides, thus regenerating thiol groups needed for the catalytic activity or the regular structure of the protein [52]. Among the proteins protected (i.e., repaired) by Trx, GR and glyceraldehyde-3-phosphate dehydrogenase (GAPDH) can be mentioned [53]. The catalytic site of TrxR has an interesting feature due to the thiol group vicinal to the selenol group, which makes this active site an attractive target for strong As(III) binding [52,54]. In a recent work by Le et al., Trx and peroxiredoxin-1 were the two most abundant proteins among the 48 identified As-binding proteins in A549 human lung carcinoma cells [55]. 2-Cys peroxiredoxins (PRXs) function as scavengers of H_2_O_2_ [56,57,58], peroxynitrite [59], and organic hydroperoxides [56], similarly to the better-known GPX. They are crucial for antioxidant and antinitrative protection in the mitochondria of various organs, including the brain [60,61]. Additionally, PRXs play a central role in human sperm physiology because they protect against oxidative stress, ensuring proper spermatozoa function and DNA integrity [62]. In other mammalian species, PRXs have also been found in oocytes, but this has not been studied in humans.

TrxR is irreversibly inhibited by arsenic trioxide or arsenite with an IC_50_ of 0.25 μM [63]. The activity of TrxR was significantly decreased in pancreatic β-cells that had been treated for 96 h with a low level (0.25–1 μM) of sodium arsenite (NaAsO_2_) [64]. Furthermore, the less toxic As(V) can be converted to As(III) intracellularly [45]. TrxR is also part of one of the two parallel chains of electron transport that go from NADPH to ribonucleotide reductase (RNR)-with the latter enzyme needed for DNA synthesis and hence for cellular growth and mitochondrial biogenesis but also NA repair [65]. For DNA repair, it will obviopecially harmful if both of the two parallel chains of electron transport going from NADPH to RNR are inhibited simultaneously. This will be the case if TrxR is inhibited by As (as well as by some other toxic agents) at the same time as GSH is depleted because of protein malnutrition or because there is a riboflavin deficiency [49,52]. The latter condition may occur among people with a high intake of polished rice since this treatment depletes the riboflavin concentration unless the rice has been fortified with vitamin B [49].

### 2.4. Selenoproteins

There are several selenoproteins other than TrxR with a structure that makes it likely that they also can be especially vulnerable targets for inhibition by arsenite, or other toxic heavy metals such as mercury (Hg), because of the formation of chelates or complexes where the toxic metal ion is bound to S and a Se atom. As examples, selenoprotein H, T, V, and W [66] can be mentioned. Selenoprotein W is an antioxidant enzyme scavenging H_2_O_2_ [67,68], expressed in nerve cells [68]. In primates, the highest levels of selenoprotein W have been found in skeletal muscle, the heart, the brain, and the tongue [69]. Selenoprotein P should also be mentioned among the proteins capable of forming selenylsulfide bonds and thus very likely chelates with toxic heavy metals, where the metal ion is simultaneously coordinated to an S atom and a Se atom [70]. This probably has not been studied how this affects the peroxynitrite and lipid hydro-peroxide-scavenging activities of selenoprotein P. Selenoprotein P is crucial for protection against atheromatosis [70]. The membrane selenoproteins K, S, T, N, and I all form selenylsulfide bonds, leading to the formation and stabilization of protein complexes required for protein trafficking [71]. Another possible example that can be mentioned is the endoplasmic reticulum-resident protein, Sep15, a thioredoxin-like member of the selenoprotein family, which may be linked to the glycoprotein folding process in cooperation with UDP-glucose: glycoprotein glucosyltransferase (UGGT) [72].

The inhibition of TrxR plus several other selenoproteins with a similar structure, making them especially vulnerable to inhibition by Hg^2+^ or arsenite, can presumably explain the different pathologies affecting multiple organs that are observed both in Hg and As poisoning.

### 2.5. Zinc-Finger Proteins

**Figure 2:**
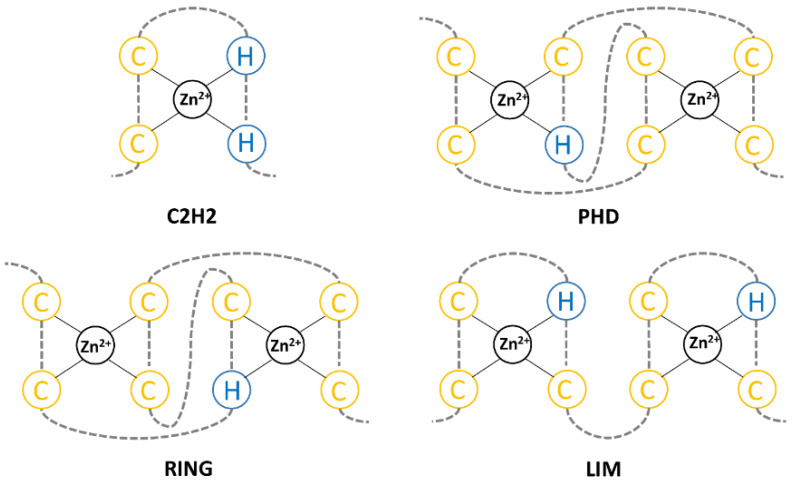
Scheme of C2H2, PHD, RING, and LIM zinc-finger domains.

Zinc-finger proteins are a broad class of proteins with a wide range of molecular functions, ranging from the development and differentiation of different tissues and genome stability to tumorigenesis, cancer progression, and metastasis formation [73]. They have a wide variety of zinc-finger domains (Figure 2), which include the most abundant C2H2, really interesting new gene (RING), plant homeodomain (PHD), and Lin-ll, Isl-1, and Mec-3 (LIM domains) [74,75].

Poly (ADP-ribose) polymerase (PARP) is a zinc-finger DNA repair protein that works as an immediate cellular response to DNA damage, playing an important role in the base excision repair (BER) and maintaining a stable genome [76]. PARP contains a C-x-C-x-H-x-C (C2HC) motif to which As(III) species could bind with good affinity [77,78]. Arsenite exposure has been reported to significantly reduce PARP activity by up to 50% enzymatic downregulation upon 10 μM arsenite exposure [79]. In another study, it was found that PARP action is inhibited in cultured HeLa cells at medium concentrations as low as 10 nM, closely matching As levels in the blood and urine of the general population [76]. Thus, PARP seems even more vulnerable to inhibition by arsenite than TrxR. The interaction of As with Zn finger proteins represents an important molecular mechanism of As co-carcinogenesis [38]. The displacement of Zn by As could result in a disruption of protein function. An example is XPA protein, which plays a central role in the nuclear excision repair (NER) pathway, but also in other important non-NER biological functions (DNA replication, recombination), with its interaction with several partners, including PARP [38,80]. 

XPA possesses a C-x-C-x-C-x-C (C4) zinc-finger motif in the globular core domain, which is essential for its function and stability and vulnerable to As. It has been demonstrated that arsenite binds to the RING finger domains of RNF20-RNF40 histone E3 ubiquitin ligase, which contain C4 and CHC2 zinc-binding sites, altering the histone epigenetic mark and impairing the repair of DNA double-strand breaks [81,82]. The mechanism of As inhibition through its binding to a DNA repair zinc-finger domain has also been identified in FANCL, a protein component of the Nuclear Core Complex (NC complex), with a crucial role in DNA interstrand crosslink repair. This lesion blocks DNA replication and transcription [83]. The erythroid transcription factor, GATA-1, which regulates red blood cell development, is an As-inhibited C4 zinc-finger protein (from the concentration of 0.1 μm), thus leading to dyserythropoiesis and an imbalance of hematopoietic differentiation [84]. Estrogen receptor-alpha (NR3A1) is a nuclear receptor activated by extrogen, with a role in regulating eukaryotic gene expression and the physiological development and function of various organ systems [85]. NR3A1 is vulnerable to As binding because of the presence in its structure of two C4 zinc-finger motifs (C-x-C-x-C-x-C-xxx-C-x-C-x-C-x-C) and the hormone binding region (containing three free sulfhydryls) [86].

## 3. Approaches to the Treatment of Diseases Caused by Toxic Effects of Arsenic

### 3.1. Nutritional Interventions in Arsenic Toxicity/Poisoning

The populations most affected by arsenic poisoning are the ones in the most economically disadvantaged conditions [3]. This may be due to inadequate dietary consumption, consisting of low protein levels and micronutrients that enhance vulnerability to As-related illnesses. Malnutrition is highly prevalent in developing countries, and many individuals are likely to be deficient in energy, protein, and micronutrients, which may affect their susceptibility to arsenicosis [3]. Several human studies have identified associations between malnourishment and developing As-caused skin lesions, skin cancer, and toxic effects ([2,15] and the references therein). Studies confirm that people with poor nutrition develop skin manifestations after drinking As-contaminated water [30,87,88]. Epidemiological studies have reported information supporting a strong correlation between As exposure and neurological dysfunction in children and adults [89,90]. Arsenic-induced cardiovascular diseases in humans are interconnected with genetic, nutritional, and environmental factors [6]. In contrast, few studies suggested that a high-fat diet magnified chronic As-induced liver injury, liver fibrogenesis, and oxidative stress in tested animals, indicating that it acts synergistically or additively in developing toxicity with As [91,92,93].

Consequently, nutritional intervention may seem to be a practical and inexpensive strategy. Nutrition enhances the process of detoxification because vitamin-rich foods, proteins, and antioxidants assist in the detoxification process. Nutrition offers protection against As’s toxic effect by two aspects: (i) methylation of As and (ii) antioxidants that protect against free radical species. Methylation is the method of detoxification through S-adenosylmethionine (SAM), which acts as a donor of methyl groups. SAM derives its methyl group from the diet.

### 3.2. Natural Compounds with an Ameliorative Effect on Arsenic Toxicity

Natural compounds and their derivatives have been used to treat oxidative stress-involved diseases for a long time [94]. Bioactive molecules have raised great interest in their potential benefits largely due to their strong antioxidant activities [95]. Krishnaiah reported in a review of the antioxidant potential of medicinal plant species that different herbs can be sources of extracts with antioxidant properties which are more effective than synthetic antioxidants. It was noted that many of them have a high content of phenolic compounds, especially flavonoids [95]. A recent report showed that 34 medicinal plants and 14 natural products exhibited significant protection against As toxicity, mostly in preclinical trials and a few in clinical studies ([27] and the references therein). Some natural compounds show ameliorative impacts on As-induced subchronic toxicity [27]. The intake of some vitamins, jaggery, fruit, and tea, as well as high levels of N-acetylcysteine glutathione, zinc, and selenium, may reduce As-induced toxicity, presumably by reducing the availability or formation of toxic monomethylated species [26,30,96,97,98,99,100].

The most potent medicinal plants for treating As toxicity, according to Mehrandish et al., are *Allium sativum*, *Curcuma longa*, *Silybum marianum*, some herbal fibers, and algae [31]. Vegetables containing organosulfur compounds are useful in clearing arsenic from the liver. An organosulfur natural compound, diallyl sulfide, found in garlic (*Allium sativum*), has been showndecreasedicity and As-induced mitochondrial toxicity in rats [101]. Cabbage (*Brassica*) in general, such as cauliflower (*Brassica oleracea* var. *botrytis*), broccoli (*Brassica oleracea* var. *italica*), and turnip (*Brassica rapa* subsp. *rapa*), are very rich sources of sulfur-containing substances [31]. These plants are also very capable of removing arsenic from the soil, and for this reason, they have also been suggested in the phytoremediation of As-contaminated soil [102]. Ogra et al. showed that *Allium sativum* could absorb arsenic (in the form of arsenate) and concentrate it in particular in its roots, joints, and leaves. Elution profiles of the metalloids in the water extracts of these garlic leaves exposed to a cultivation medium containing arsenate showed that some of the accumulated As was metabolized in the reduced and toxic arsenite and other forms not clearly deciphered but presumably related to some complexed or methylated arsenic species [103]. The consumption of these plants after their use in phytoremediation represents a significant potential risk for human health in addition to the other As-contaminated foods already described [104].

The ameliorative effects of herbal extracts against in vivo experimentally induced As toxicity were evaluated for crude extracts of *Viscum album* and *Allium sativum* [105], *Moringa oleifera* leaves [106], *Syzygium cumini* leaves [107], *Phyllanthus emblica* leaves [108], and *Ipomea aquatica* aerial parts [109]. The antioxidant effects of *Lamiaceae* family representatives were proved experimentally for *Ocimum sanctum* leaf extract [110], *Mentha piperita* leaf extract [111], and essential oils of the aerial parts from *Monarda fistulosa* and *Satureja hortensis* [112]. Green tea (*Camellia sinensis*) showed a chemopreventive effect for arsenic-H_2_O_2_-related oxidant stress in vitro [113]. Research has shown that the extract from *Prunus domestica* leaves showed the highest antioxidant activity at a concentration of 2 mg/g and reduced the level of peroxidation products by an average of 88.1% for 20 min, thus proving more effective than α-tocopherol [114].

The administration of modified citrus pectin showed a significant (130%) increase in the urinary excretion of As [115]. Polyphenol-rich apple (*Malus domestica*) peel extract attenuates arsenic trioxide-induced cardiotoxicity in h9c2 cells via its antioxidant activity [116]. Extract of *Spirulina* (*Cyanobacteria*) can remove As from isolated liver tissues [117]. Flavonolignan, silibinin, present in *Silybum marianum*, has positive impacts in As-exposed rats, which are attributed to its antioxidant potential [118]. Clinical trials of arsenicosis patients in Bangladesh showed the usefulness of such antioxidants as vitamins A, C, and E [25].

**Figure 3:**
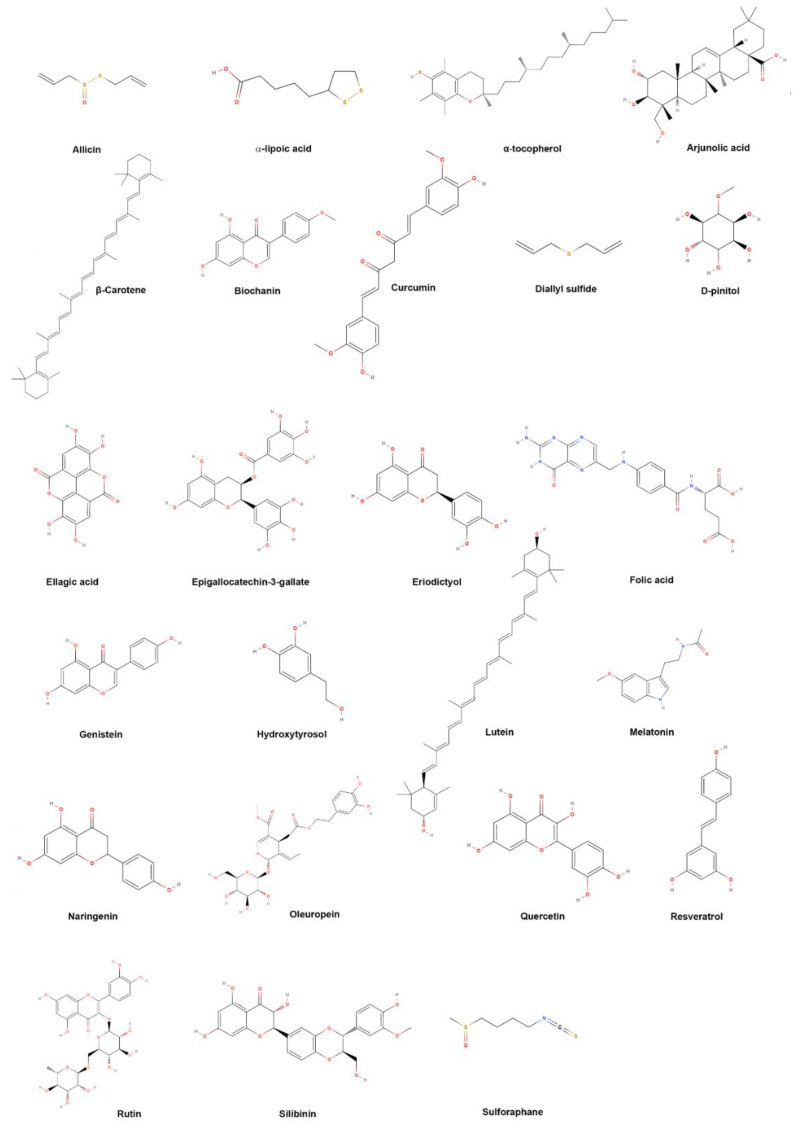
Chemical structures of the compounds active in alleviating arsenic toxicity.

Several natural compounds have been identified as active in alleviating arsenic toxicity (Figure 3, Table 1).

These include alpha-lipoic acid (α-LA) and its reduced form dihydrolipoic acid (DHLA) [23,119,120], naringenin [121], epigallocatechin-3-gallate [122,123,124], sulforaphane [125,126,127], allicin [128], eriodictyol [129], hydroxytyrosol [130], lutein [131], oleuropein [132], ellagic acid [133], curcumin [134], biochanin [135], resveratrol [136,137,138], β-Carotene [139], genistein [140], quercetin [141], rutin [142], α-Tocopherol [143,144], and D-pinitol [145], which have shown ameliorative effects against various As-induced toxicities in animal models and in vitro studies. Folic acid supplementation can decrease blood As concentrations [146]. Tetrahydrocurcumin (a metabolite of curcumin) exhibits mainly the same pharmacological effect as curcumin. Its administration showed the significant reversal of As-induced toxicity in hepatic cells [147]. Triterpenoid arjunolic acid demonstrates a protective role against As-induced cardiac oxidative damage. The free radical scavenging activity and the effect of arjunolic acid on the antioxidant power were determined from its 2,2-diphenyl-1-picryl hydrazyl radical scavenging ability and a Fe reducing/antioxidant power assay [148]. Lin and colleagues reported that a natural dietary compound, Melatonin, found in fruits, vegetables, and grains, has been found to scavenge free radicals and promote the synthesis of the glutathione peroxidase enzyme to counter oxidative stress in the brain tissue of As-administered animals [149].

Aromatherapy is a complementary and preventive medical practice that uses essential oils as the major therapeutic agents to treat many diseases [150]. Essential oils can improve the function of the immune system, activate the receptors of the skin and respiratory system, etc. [112,150]. Inhalation, local application, and baths are the major methods used in aromatherapy that utilize these oils to penetrate the human skin. Essential oils can modulate the body systems’ activities [150]. Oil from the *Allium sativum* bulb was also effective in the improvement of As-induced keratosis [151].

### 3.3. Selenium

Selenium (Se), an essential micronutrient owing to its antioxidant and antagonistic characteristics, is a potential mitigator of As toxicity [152,153]. The protective effects of selenium, calcium, and magnesium against As-induced oxidative stress in male rats have been studied. The results of these studies indicated favorable effects on hematological and other biochemical parameters by all three elements. Still, selenium was the most effective in lessening As poisoning compared to the others [154]. Messarah et al. suggested that As exposure enhanced oxidative stress by disturbing the tissue antioxidant defense system. Still, the Se coadministration protected liver tissues against As intoxication, probably owing to its antioxidant properties [155]. High-Se lentils can potentially mitigate As toxicity in rats [156]. In a randomized, double-blind, placebo-control trial in Bangladesh, higher dietary selenium increased urinary arsenic excretion over six months and offered relief against chronic arsenic poisoning [157].

Brazil nuts contain high amounts of Se and can be a good strategy for detoxifying As poisoning. The content of organic Se (mainly as selenomethionine) in Brazil nuts varies from 2.7 to 11 mg Se/g [158]. Thus, Mazokopakis and Liontiris concluded that two to seven Brazil nuts met the daily Se requirement [159]. Lima et al. studied the content of Se and its localization in nuts and found that average Se levels ranged from 28 to 49 mg/kg, with an 8-fold difference in seed content [160]. The highest concentration was in the ring 1–2 mm below the surface of the nut. Consumption of one seed (5 g) from high Se content nuts corresponds to the recommended daily intake [160].

The pool of organic selenides, in addition to the abovementioned selenomethionine in Brazil nuts, includes selenocysteine, selenate, selenite, and c-glutamyl methylselenocysteine, found in *Brassica* and *Allium* vegetables [158]. Plessi et al. found that the Se content in the edible portion of commercial fishes ranges from a minimum of 0.134 mg/kg (halibut, *Hippoglossus hippoglossus*) to a maximum of 0.734 mg/kg (tuna, *Thunnus thynnus*), with the average value being 0.307 mg/kg of fresh weight [161]. The Se from organic selenides is absorbed and, according to metabolic pathways, either used for selenoprotein synthesis or excreted in the urine as a selenosugar [158]. Selenomethionine is also the major form in meat (for example, lamb contains 0.4 mg/kg of fresh weight) [158]. Bügel et al.’s research showed that most of the Se was absorbed from meat, and over half was retained in the body [162]. The protective effects of Se on oxidative damage induced by sodium arsenite in rat liver were determined. It was found that Se protects liver cells by adjusting the expression of oxidative stress-related genes to improve the activities of antioxidant enzymes [163]. In 2011, Pilsner et al. reported a result that suggested that plasma Se may reduce the body’s burden of As and help to minimize the concentration of the most toxic metabolites, MMA^V^ to MMA^III^, of the As methylation pathway [164].

### 3.4. Zinc

Zinc is the main source of antioxidants, and it acts by two mechanisms: (i) it protects sulfhydryl groups against oxidation, and (ii) it inhibits the production of reactive oxygen by transition metals [73]. It has been reported that the administration of zinc reduces the As-induced teratogenic effect, reduces acute As toxicity in rats by reinstating antioxidant activity, increases metallothionein expression independently, and reduces oxidative stress in kidney tissue by decreasing malondialdehyde and increasing glutathione levels [165,166,167]. In a comparative analysis of the zinc content in the raw materials of plants of the *Rosaceae* family, namely the leaves of almonds, cherries, plums, apricots, and peaches, as well as in their shoots, buds, and bark, it was found that almond leaves and plum leaves were on top in Zn content, exceeding the content of this element in other raw materials by 5–20 times [168]. Therefore, these plants might be promising sources of natural extracts with high Zn content.

## 4. Concluding Remarks

There is no particular therapy for chronic exposure to As due to long-term ingestion of contaminated water and food. Diet and nutrition can affect As's bioaccessibility, metabolism, and toxicity. Micronutrient deficiency in foods can lead to more pronounced toxicity of As. Arsenic has the capacity to imbalance antioxidant homeostasis by the generation of reactive oxygen species in mammalian tissues. Thus, therapeutic strategies that could lead to the increased antioxidant capacity of cells could reinforce long-term efficient As poisoning intervention. This can be achieved by using exogenous supplementation of antioxidant molecules to support antioxidant defenses in cells. Therefore, nutritional antioxidants remove active oxygen and scavenge free radical species and repair the oxidized membranes of the cells. Polyphenols, flavonoids, amino acids, protein, and functional foods such as jaggery and honey are supplements that can be useful in the fight against As toxicity.

Many natural dietary compounds and proper nutrition exhibit a better prophylactic effect than a therapeutic effect against As-mediated toxicity. Therefore, such types of natural compounds and adequate nutrition can be used as a dietary supplement to prevent any adverse effects that may occur due to As intoxication or as an adjuvant along with chelators for treating As-induced toxicity. Nutritional combination therapy is more useful in managing chronic As toxicity than usual chelation monotherapy. An extensive clinical study is needed to accurately determine the dose of nutraceuticals and functional foods against As toxicity. Studies on the possible protective roles of selenol and thiol compounds, including new chelators against toxic As species, are of great importance in the future.

The review findings encourage further mechanistic preclinical and appropriately designed clinical studies on natural dietary compounds, proper nutrition, and natural products, especially in managing human chronic As toxicity. The field remains open to exploring phytochemicals, natural compounds, and proper nutrition formulations that can not only offer protection against As-mediated toxicity but can also serve as therapeutic formulations to reverse the toxic effects of As.

## Figures and Tables

**Table 1:** Natural compounds active in alleviating arsenic toxicity.

Common Name	IUPAC Name
Allicin	3-prop-2-enylsulfinylsulfanylprop-1-ene
α-Lipoic acid	5-(dithiolan-3-yl)pentanoic acid
α-Tocopherol	(2*R*)-2,5,7,8-tetramethyl-2-[(4*R*,8*R*)-4,8,12-trimethyltridecyl]-3,4-dihydrochromen-6-ol
Arjunolic acid	(4a*S*,6a*R*,6a*S*,6b*R*,8a*R*,9*R*,10*R*,11*R*,12a*R*,14b*S*)-10,11-dihydroxy-9-(hydroxymethyl)-2,2,6a,6b,9,12a-hexamethyl-1,3,4,5,6,6a,7,8,8a,10,11,12,13,14b-tetradecahydropicene-4a-carboxylic acid
Biochanin	5,7-dihydroxy-3-(4-methoxyphenyl)chromen-4-one
β-Carotene	1,3,3-trimethyl-2-[(1*E*,3*E*,5*E*,7*E*,9*E*,11*E*,13*E*,15*E*,17*E*)-3,7,12,16-tetramethyl-18-(2,6,6-trimethylcyclohexen-1-yl)octadeca-1,3,5,7,9,11,13,15,17-nonaenyl]cyclohexene
Curcumin	(1*E*,6*E*)-1,7-bis(4-hydroxy-3-methoxyphenyl)hepta-1,6-diene-3,5-dione
Diallyl sulfide	3-prop-2-enylsulfanylprop-1-ene
D-pinitol	(1*S*,2*S*,4*S*,5*R*)-6-methoxycyclohexane-1,2,3,4,5-pentol
Ellagic acid	6,7,13,14-tetrahydroxy-2,9-dioxatetracyclo[6.6.2.04,16.011,15]hexadeca-1(15),4,6,8(16),11,13-hexaene-3,10-dione
Epigallocatechin-3-gallate	[(2*R*,3*R*)-5,7-dihydroxy-2-(3,4,5-trihydroxyphenyl)-3,4-dihydro-2H-chromen-3-yl] 3,4,5-trihydroxybenzoate
Eriodictyol	(2*S*)-2-(3,4-dihydroxyphenyl)-5,7-dihydroxy-2,3-dihydrochromen-4-one
Folic acid	(2*S*)-2-[[4-[(2-amino-4-oxo-3*H*-pteridin-6-yl)methylamino]benzoyl]amino]pentanedioic acid
Genistein	5,7-dihydroxy-3-(4-hydroxyphenyl)chromen-4-one
Hydroxytyrosol	4-(2-hydroxyethyl)benzene-1,2-diol
Lutein	(1*R*)-4-[(1*E*,3*E*,5*E*,7*E*,9*E*,11*E*,13*E*,15*E*,17*E*)-18-[(1*R*,4*R*)-4-hydroxy-2,6,6-trimethylcyclohex-2-en-1-yl]-3,7,12,16-tetramethyloctadeca-1,3,5,7,9,11,13,15,17-nonaenyl]-3,5,5-trimethylcyclohex-3-en-1-ol
Melatonin	*N*-[2-(5-methoxy-1*H*-indol-3-yl)ethyl]acetamide
Naringenin	5,7-dihydroxy-2-(4-hydroxyphenyl)-2,3-dihydrochromen-4-one
Oleuropein	methyl (4*S*,5*E*,6*S*)-4-[2-[2-(3,4-dihydroxyphenyl)ethoxy]-2-oxoethyl]-5-ethylidene-6-[(2*S*,3*R*,4*S*,5*S*,6*R*)-3,4,5-trihydroxy-6-(hydroxymethyl)oxan-2-yl]oxy-4*H*-pyran-3-carboxylate
Quercetin	2-(3,4-dihydroxyphenyl)-3,5,7-trihydroxychromen-4-one
Resveratrol	5-[(*E*)-2-(4-hydroxyphenyl)ethenyl]benzene-1,3-diol
Rutin	2-(3,4-dihydroxyphenyl)-5,7-dihydroxy-3-[(2*S*,3*R*,4*S*,5*S*,6*R*)-3,4,5-trihydroxy-6-[[(2*R*,3*R*,4*R*,5*R*,6*S*)-3,4,5-trihydroxy-6-methyloxan-2-yl]oxymethyl]oxan-2-yl]oxychromen-4-one
Sibilin	(2*R*,3*R*)-3,5,7-trihydroxy-2-[(2*R*,3*R*)-3-(4-hydroxy-3-methoxyphenyl)-2-(hydroxymethyl)-2,3-dihydro-1,4-benzodioxin-6-yl]-2,3-dihydrochromen-4-one
Sulforaphane	1-isothiocyanato-4-methylsulfinylbutane

## Abbreviations

As	arsenic
BAL	British Anti-Lewisite
CAT	catalase
iAsIII	inorganic As(III)
MeAs(OH)2, MMAIII	monomethylarsonous acid
Me2AsOH, DMAIII	dimethylarsinous acid
NADPH	nicotinamide adenine dinucleotide phosphate
GPX	glutathione peroxidases
GR	glutathione reductase
GSH	glutathione
GSSG	glutathione disulfide
GSTs	glutathione S-transferase
PARP	poly (ADP-ribose) polymerase
PDH	pyruvate dehydrogenase
SAM	S-adenosylmethionine
Se	Selenium
SOD	superoxide dismutase
Trx	thioredoxin
TrxR	thioredoxin reductase
